# Comparison of Recovery Quality Following Medetomidine versus Xylazine Balanced Isoflurane Anaesthesia in Horses: A Retrospective Analysis

**DOI:** 10.3390/ani11082440

**Published:** 2021-08-19

**Authors:** Isabel Kälin, Inken S. Henze, Simone K. Ringer, Paul R. Torgerson, Regula Bettschart-Wolfensberger

**Affiliations:** 1 Section of Anaesthesiology, Vetsuisse Faculty, University of Zurich, 8057 Zurich, Switzerland; ihenze@vetclinics.uzh.ch (I.S.H.); sringer@vetclinics.uzh.ch (S.K.R.); rbettschart@vetclinics.uzh.ch (R.B.-W.); 2 Section of Epidemiology, Vetsuisse Faculty, University of Zurich, 8057 Zurich, Switzerland; paul.torgerson@access.uzh.ch

**Keywords:** equine, alpha-2 adrenergic agonist, PIVA

## Abstract

**Simple Summary:**

Recovery from general anaesthesia poses the most critical phase of equine anaesthesia and is the main cause for the relatively high anaesthetic mortality rate compared to other species. It is, therefore, essential to identify anaesthetic protocols that promote safe recoveries. This retrospective study compared the quality of 470 recoveries following general anaesthesia with the anaesthetic gas isoflurane combined with a constant rate infusion of two different alpha-2 adrenergic agonists (xylazine or medetomidine). On the basis of video recordings, recovery quality was scored by two observers unaware of animal details, procedure, or drugs used. Additionally, factors that may affect recovery (e.g., breed, age, procedure, duration of anaesthesia, and intraoperative complications) were taken into consideration. Horses needing higher doses of xylazine to sedate prior to anaesthesia, the intraoperative use of tetrastarch for cardiovascular support, and the use of salbutamol to improve inadequate blood oxygenation during general anaesthesia were related to poorer recovery scores. Whilst recoveries of horses treated with medetomidine took significantly longer compared to xylazine, the attempts to stand and the overall quality of recovery were similar for both groups, indicating that both anaesthetic protocols promote similarly safe recoveries.

**Abstract:**

Medetomidine partial intravenous anaesthesia (PIVA) has not been compared to xylazine PIVA regarding quality of recovery. This clinical retrospective study compared recoveries following isoflurane anaesthesia balanced with medetomidine or xylazine. The following standard protocol was used: sedation with 7 µg·kg^−1^ medetomidine or 1.1 mg·kg^−1^ xylazine, anaesthesia induction with ketamine/diazepam, maintenance with isoflurane and 3.5 µg·kg^−1^·h^−1^ medetomidine or 0.7 mg·kg^−1^·h^−1^ xylazine, and sedation after anaesthesia with 2 µg·kg^−1^ medetomidine or 0.3 mg·kg^−1^ xylazine. Recovery was timed and, using video recordings, numerically scored by two blinded observers. Influence of demographics, procedure, peri-anaesthetic drugs, and intraoperative complications (hypotension, hypoxemia, and tachycardia) on recovery were analysed using regression analysis (*p* < 0.05). A total of 470 recoveries (medetomidine 279, xylazine 191) were finally included. Following medetomidine, recoveries were significantly longer (median (interquartile range): 57 (43–71) min) than xylazine (43 (32–59) min) (*p* < 0.001). However, the number of attempts to stand was similar (medetomidine and xylazine: 2 (1–3)). Poorer scores were seen with increased pre-anaesthetic dose of xylazine, intraoperative tetrastarch, or salbutamol. However, use of medetomidine or xylazine did not influence recovery score, concluding that, following medetomidine–isoflurane PIVA, recovery is longer, but of similar quality compared to xylazine.

## 1. Introduction

Despite ongoing advancements and improvements in equine anaesthetic management, recovery poses the most critical phase of equine general anaesthesia, causing a relatively high anaesthetic mortality rate compared to other species [1], with arguably the most representative study to date reporting an overall mortality rate of 1.9% and 0.9% after exclusion of emergency abdominal surgeries [2].This is reflected in the continuous effort in veterinary research to find and understand factors affecting recovery outcome and reducing mortality rate [3]. For surgical interventions requiring prolonged anaesthesia, volatile anaesthetic agents, such as isoflurane, are commonly used. However, isoflurane causes dose-dependent cardiovascular depression and does not provide analgesia [4]. The cardiovascular side-effects might lead to poor perfusion and tissue hypoxia, resulting in post-anaesthetic myopathy and neuropathy [5,6,7], which can be detrimental to the recovery phase. To provide intraoperative analgesia and to reduce the minimal alveolar concentration of isoflurane, partial intravenous anaesthesia (PIVA) is routinely performed in horses, most commonly using either lidocaine, ketamine, alpha-2 adrenergic agonists, or a combination thereof [8,9].

Various alpha-2 adrenergic agonists at differing dose rates have been used for PIVA in horses [10,11,12,13,14]. Following bolus administration of alpha-2 adrenergic agonists, typical untoward side-effects, such as an increase in arterial blood pressure caused by peripheral vasoconstriction, followed by bradycardia, are reported [15]. Conversely, during constant rate infusion (CRI) of medetomidine and xylazine, these effects are mitigated, and, at steady-state plasma levels, cardiopulmonary depression is minimal [16,17]. Both drugs are used for PIVA in horses and were compared in a recent study by Wiederkehr et al., who observed a quicker recovery from anaesthesia following xylazine PIVA as opposed to medetomidine PIVA [18]. However, the study concluded that the investigation of a greater number of recoveries was necessary to detect any significant difference in recovery quality between the two PIVA regimes.

The goal of the present study was to assess retrospectively, using video recordings, the quality of recovery of horses undergoing elective and emergency surgery under isoflurane general anaesthesia concurrently with xylazine or medetomidine CRI. The hypothesis was that the incidence of poor-quality and potentially dangerous recoveries following medetomidine PIVA is not different from xylazine PIVA.

## 2. Materials and Methods

In the present study, video recordings of horses recovering from general anaesthesia were retrospectively evaluated. Owner consent for the use of their horse’s data and recordings, as part of a general consent form, was obtained upon admission at the Equine Clinic of the Vetsuisse Faculty of the University of Zurich (hereafter referred to as “the clinic”).

### 2.1. Case Selection

On the basis of previous in-house publications, it was assumed that the incidence of bad recoveries (defined as scores of 4 or 5 on our in-house recovery scoring system of 1–5) in our population treated with xylazine was 12% [18]. A power analysis revealed that 496 comparable recoveries would be necessary to detect if medetomidine led to a reduction in the incidence of bad recoveries to 5% (α = 0.05, power 0.8). Data and videos were collected from horses undergoing general anaesthesia at the clinic between October 2017 and October 2019. Inclusion criteria were any horses older than 3 months and heavier than 200 kg with complete anaesthetic records. Exclusion criteria were Equidae other than horses (i.e., mules or donkeys), horses undergoing total intravenous anaesthesia or PIVA with an alpha-2 adrenergic agonist other than xylazine or medetomidine, and horses with a preoperative history of ataxia.

### 2.2. Anaesthetic Protocol and Monitoring

All horses underwent a pre-anaesthetic general examination and were given a physical status American Society of Anaesthesiologists (ASA-score) by the anaesthetist in charge. To allow for exact drug administration, all horses were weighed prior to anaesthesia. Horses undergoing elective surgical procedures were starved for 8–12 h before anaesthesia, whilst access to water was provided at any time. For intravenous (IV) administration of drugs, a jugular venous catheter was placed at least 45 min prior to sedation for anaesthesia.

Premedication with penicillin Na^+^ (Penicillin Natrium Streuli ad us. vet.; Streuli Pharma AG, Uznach, Switzerland; 30,000 IU·kg^−1^), gentamicin (Genta 10%; CP-Pharma, Burgdorf, Germany; 10 mg·kg^−1^), and either flunixin (Fluniximin ad us. vet.; Graeub AG, Bern, Switzerland; 1.1 mg·kg^−1^) or phenylbutazone (Butadion ad us. vet.; Streuli Pharma AG; 4 mg·kg^−1^) was administered IV 30–45 min prior to anaesthesia. Given no contraindications, such as expected intraoperative blood loss or compromised cardiovascular state, acepromazine (Prequillan ad us vet.; Arovet AG, Dietikon, Switzerland; 0.03 mg·kg^−1^) was administered intramuscularly (IM) at the same time as the antibiotics. On the way to the operating theatre, the horses’ mouths were routinely flushed with water. Before induction of anaesthesia, horses were sedated with either xylazine (Xylazin 2%; Streuli Pharma AG; 1.1 mg·kg^−1^) or medetomidine (Medetor ad us. vet.; Virbac AG, Opfikon, Switzerland; 7 µg·kg^−1^) IV. In the country where the current study took place, only non-food-producing animals can receive medetomidine; therefore, the decision to treat with either xylazine or medetomidine was based on the official registration status of the horse (i.e., food-producing or non-food-producing animal, respectively). Horses with unknown status received xylazine.

At the anaesthetist in charge’s discretion, the dose of alpha-2 adrenergic agonist was adapted to produce sedation considered sufficient for anaesthesia induction (e.g., lowered head, no reaction when being approached, knuckling, and indifference to surroundings). Induction of general anaesthesia was achieved with ketamine (Ketanarkon 100 ad us. vet.; Streuli Pharma AG; 2.2 mg·kg^−1^) and diazepam (Valium 10 mg; Roche Pharma AG, Basel, Switzerland; 0.02 mg·kg^−1^) IV. Once the horses were recumbent, the trachea was intubated, and the horse was hoisted onto a horse surgery table and connected to a large animal anaesthetic machine (Mallard 2800C-P; Mallard Medical/AB Medical Technologies Inc., Redding, CA, USA or Tafonius; Hallowell Engineering & Manufacturing Corp., Pittsfield, MA, USA.). In all horses, to monitor urinary output and avoid overfilling of the bladder, a urinary catheter was placed immediately after anaesthesia induction and kept in place until recovery was complete.

Anaesthesia was maintained using isoflurane (IsoFlo; Provet AG, Lyssach, Switzerland) in oxygen and air (initial inspired fraction of oxygen (FiO_2_) 0.45–0.55, increased up to 1.0 to maintain arterial oxygen saturation (SaO_2_) > 90% or arterial partial pressure of oxygen (PaO_2_) > 80 mmHg). Salbutamol (Ventolin; GlaxoSmithKline AG, Zug, Switzerland; 2 µg·kg^−1^) was administered intratracheally if PaO_2_ fell below 60 mmHg. Horses were allowed to breathe spontaneously. Artificial ventilation was initiated if, within 5–10 min of anaesthesia induction, no regular spontaneous respiration could be established, apnoea phases longer than 45 s occurred, or end-tidal CO_2_ (ETCO_2_) > 70 mmHg was recorded.

General anaesthesia was maintained with isoflurane (titrated to maintain a sluggish palpebral reflex) and a CRI of either xylazine (0.7 mg·kg^−1^·h^−1^) or medetomidine (3.5 µg·kg^−1^·h^−^^1^). If the anaesthetic depth was insufficient, a single IV bolus of ketamine (0.1–0.2 mg·kg^−1^) in the case of nystagmus or thiopental (Thiopentalum natricum; Ospedalia AG, Hünenberg, Switzerland; 0.5–1 mg·kg^−1^) in the case of movement was administered and noted on the anaesthetic protocol. If additional analgesia was required (judged by the anaesthetist in charge), an IV bolus of lidocaine (Lidocain Streuli ad us. vet.; Streuli Pharma AG; 1–2 mg·kg^−1^), followed by a CRI (1.5–3 mg·kg^−1^·h^−1^) was administered; alternatively, a CRI of ketamine (0.6 mg·kg^−1^·h^−1^) was initiated. The use of local anaesthesia (using local anaesthetics) or local analgesia (administering opioids locally, for example, epidurally or intraarticularly) techniques was noted.

Standard monitoring during anaesthesia included judgement of presence of nystagmus and palpebral reflex, colour of mucous membranes and capillary refill time, capnography (ETCO_2_, ET isoflurane, FiO_2_), respiratory rate, tidal volume, pulse rate, ECG, invasive arterial blood pressures, arterial blood gas analysis (performed every 30–60 min with a Co-oxymeter, RAPIDPoint^®^500; Siemens, München, Germany), pulse oximetry, urinary output, and body temperature. All parameters were recorded every 5 min.

All horses initially received a CRI of lactated Ringer’s solution at a starting rate of 10 mL·kg^−1^·h^−1^, as well as a CRI of dobutamine (Dobutrex; Teva Pharma AG, Basel, Switzerland) at an initial rate of 30 µg·kg^−1^·h^−^^1^. Once invasive blood pressure measurement was available, the rate of dobutamine was continuously adapted to maintain a mean arterial blood pressure (MAP) between 70 and 90 mmHg. Throughout anaesthesia, the CRI of lactated Ringer’s solution was continuously adapted to the horses’ cardiovascular state, estimated blood loss, and urinary output.

Horses with suspected endotoxaemia received polymyxin B (Polymyxin B Sulphate 100,000 IE·mL^−1^; Kantonsapotheke Zurich, Zurich, Switzerland; 6000 IU·kg^−1^) diluted in 500 mL of isotonic saline slowly IV (over 30 min) and/or hydrocortisone if endotoxaemic shock was anticipated or prevalent (Solu-CORTEF 500 mg; Pfizer AG, New York City, NY, USA; 1–4 mg·kg^−1^). In horses undergoing laparotomies or receiving bone implants, penicillin was repeated intraoperatively every 2 h. Then, 20–40 min before the end of surgery, all horses were administered morphine (0.1 mg·kg^−1^) IM.

At the end of anaesthesia, phenylephrine 0.15% (Phenylephrini hydrochloridum; Streuli Pharma AG 0.03 mg·kg^−1^) was bilaterally administered intranasally in the ventral meatus. Before being moved to a padded recovery box heated with infrared lamps, a padded helmet was fitted to each horse’s head. Horses were supplied with oxygen flow-by (15 L per minute) in the ET tube or, after extubation, intranasally. For recovery, all horses received IV sedation upon arrival in the recovery box with either 0.3 mg·kg^−1^ xylazine or 2 µg·kg^−1^ medetomidine. At the discretion of the anaesthetist in charge, the dose of sedation was increased and noted. Extubation was routinely performed 10–15 min after the end of isoflurane administration; horses undergoing emergency laparotomy surgeries or emergencies that had not been starved were only extubated in the presence of a swallowing reflex. Generally, horses were left to recover freely, but they were assisted with ropes or manually for certain indications (e.g., caesarean section, geriatric horse, horse with cast) by the on-duty personnel. For rope recovery, one rope was attached to the neck piece of the helmet and a second rope was tied to the tail. Each rope was operated by one person. Then, the horses were left undisturbed. Horses’ ears were plugged with cotton wool to avoid acoustic stimulation. If the horse was still in lateral recumbency after 60 min, it was stimulated by noise and touch to get into a sternal position. If the horse failed to move, further assessment was made to determine whether the horse should receive additional fluids or glucose-containing fluids (dependent on whether the horse was judged hypovolaemic or exhausted).

### 2.3. Video Analysis of Recoveries

As per standard protocol, all recoveries at the clinic are recorded on DVD with a camera installed on the ceiling of the recovery boxes. Analysis of the videos was done by scorers blinded to treatment, type, and duration of surgery. The timing of recovery (i.e., time spent in lateral recumbency, time spent in sternal recumbency, and time until standing), number of attempts to get into a sternal position, and number of attempts to stand were assessed on the basis of the video recordings and noted by the principal investigator. If the horse went back and forth between lateral and sternal recumbency, the added time spent in each recumbency was recorded. In order to prevent scorer fatigue, the recordings were edited by the principal investigator to exclude phases of prolonged inactivity. The clips were given to two scorers (scorer 1: principal investigator, i.e., a second-year equine anaesthesia intern; scorer 2: a second-year anaesthesia resident). Table 1 describes the in-house numerical recovery quality scoring system (RQSS) which was used to assess the quality of recovery.

The agreement between scorers 1 and 2 was assessed. If the scorers scored the same horse with a difference greater than 2, or if one scorer allocated a score of 3 and the other a score of 4, the clips were given to two additional scorers (scorers 3 and 4, both > 10 years of experience as ECVAA diplomates) for assessment.

### 2.4. Animal Data

Each horse’s data were automatically compiled from the clinic record system into a spreadsheet using Microsoft Excel. Information missing in the electronic database was manually retrieved from the clinic system and from the anaesthetic protocols. The following demographic horse data were collected: age, weight, sex (stallion, mare, or gelding), breed, and ASA score. Breeds were grouped into warmblood (e.g., Swiss warmblood, Hanoverian, Oldenburger, and Irish sports horse), Thoroughbred, Arabian (including Thoroughbred Arabian, Partbred Arabians, Shagya-Arabian, and Pintarabian), Baroque horse (such as Kladruber, Andalusian, Friesian, Lippizan, Lusitano, Menorquín horse, and Knabstrup horse), American horse (such as Quarter Horse and Appaloosa), Icelandic horse, pony, draught horse (e.g., Shire horse and Noriker), small draught horse (e.g., Haflinger, Swiss mountain horse, and Fjord horse), and unknown. Furthermore, it was noted if the horse underwent general anaesthesia within the last 6 months of the recovery judged.

As for general information on the procedure, it was noted if the surgery was an emergency or not. The type of the procedure was categorised into seven groups: laparotomy (e.g., emergency colic surgeries), orthopaedic surgery, orthopaedic limb fracture repair, ophthalmologic surgery, visceral surgery (e.g., castration, hernia repair, skin tumour removal), and surgeries on the head and throat (e.g., tie-back, sinus flaps, dental procedures); the seventh group contained horses that could not be clearly allocated to one of the aforementioned groups because they underwent several procedures. Positioning of the horse during surgery was described as either dorsal, right- or left-lateral recumbency, or changing position. It was noted if the horse was breathing spontaneously, was mechanically ventilated, or was partially mechanically ventilated (i.e., started with spontaneous breathing but had to be switched to mechanical ventilation). Special requirements for the surgical intervention, such as use of tourniquet or neuromuscular blocking agents (NMBA), were also documented. The table also included if and which local analgesia or anaesthesia the horse received, and which drug was used. If a tourniquet was applied, the duration was noted. The duration of the anaesthetic time (time from induction to end of isoflurane) was routinely documented by the anaesthetist in charge and included for the statistical analysis of this study.

It was further classified whether the anaesthetic protocol was based on xylazine (group XYL) or medetomidine (group MED). The dose of the respective alpha-2 adrenergic agonist for sedation prior to induction and for post-sedation during recovery was noted. Information about the peri-anaesthetic use of the following drugs was documented: use and dose of acepromazine; administration of ketamine and thiopental boli, represented as total dose in mg·kg^−1^·h^−1^; use of a lidocaine or ketamine CRI including duration of administration, dose per kg bodyweight per hour, total dose, and time from end of CRI to end of anaesthesia; administration and total dose of tetrastarch (mL·kg^−1^); the use of further drugs deviating from the standard protocol (e.g., additional analgesics, single lidocaine bolus, salbutamol, hydrocortisone, polymyxin B, tranexamic acid, and hypertonic saline).

Anaesthetic events considered relevant for the quality of recovery, i.e., the occurrence of hypotension, tachycardia and hypoxaemia, were included in the spreadsheet. A horse was classified as hypotensive if the MAP was below 70 mmHg for at least three consecutive 5 min readings. Horses with only non-invasive blood pressure measurements were marked in order to differentiate from the invasive measurements. The total duration of hypotension and the range of mean arterial blood pressure values were noted. Tachycardia was defined as a heart rate > 50 beats per minute. The duration of tachycardia and range of the heart rate were noted. A horse was considered hypoxaemic if PaO_2_ in the arterial blood gas fell below 60 mmHg. PaO_2_ values between 60 and 80 mmHg were classified as “mildly hypoxaemic”. If several blood gas analyses were available, the allocation to “hypoxaemic” vs. “mildly hypoxaemic” was based on the lowest PaO_2_ reading. If no arterial blood gas was available, the pulse oximetric readings were reviewed. A horse was classified as hypoxaemic if the pulse oximetry values were <90%.

Regarding recovery, the only information that was taken from the clinic system and/or anaesthetic protocol was whether the horse recovered with an endotracheal (ET) tube in place and whether the recovery was assisted or not. If a horse was only assisted after initial unsuccessful attempts to stand on its own, it was categorised as “non-assisted” with a note that it ultimately stood up with support.

### 2.5. Statistical Analysis

Statistical analyses were performed using R 4.0.2 (R Core Team (2020); R: A language and environment for statistical computing. R Foundation for Statistical Computing, Vienna, Austria; URL https://www.R-project.org/, accessed on 10 September 2020) and figures were produced using GraphPad PRISM 9 (GraphPad Software, San Diego, CA, USA). First, normality of data distribution was assessed using a Kolmogorov–Smirnov test for age, weight, duration of anaesthesia, time in lateral recumbency and sternal position, time to stand, attempts to achieve sternal position and to stand, and drug dose (for acepromazine, tetrastarch, and boli of lidocaine, thiopental, and ketamine).

Accordingly, both groups (XYL and MED) were compared using either a Student’s *t*-test for parametric data or a Mann–Whitney U-test for non-parametric data. Recoveries were allocated into a dichotomous system (good: scores 1–3; bad: scores 4–5) to perform a linear regression analysis to evaluate factors affecting recovery quality. Investigated factors included use and dose of xylazine or medetomidine, times spent in lateral and sternal recumbency, time until standing, assisted recovery, recovery with ET tube, bodyweight, age, breed, sex, ASA score, procedure, repeated surgery, emergency, positioning, ventilation mode, administration of additional drugs (i.e., tetrastarch, salbutamol, polymyxin B, ketamine, thiopental, lidocaine, NMBA, local anaesthesia, and analgesia), duration of anaesthesia, use of a tourniquet, hypotension, hypoxaemia, and tachycardia during anaesthesia. Data were further analysed using mixed-effects ordinal regression for the numerical recovery scores (1–5) with scorer and the other factors as fixed effects, whilst horse was a random effect to assess if scorer 1 and scorer 2 gave similar results and to identify factors affecting recovery quality. Finally, kappa statistics were used to determine interrater agreement between scorer 1 and scorer 2. Depending on data distribution, results are either presented as mean ± SD or median (range). Significance was considered for a *p*-value < 0.05.

## 3. Results

A total of 496 recoveries from 466 horses and ponies fulfilled the criteria to be included in this study and were scored. Of these, 28 horses were anaesthetised twice, and one horse was anaesthetised three times. After being scored by scorers 1 and 2, 56 recoveries had to be reassessed by scorers 3 and 4. In 26 instances (XYL 11; MED 15), fewer than three scorers agreed; therefore, these cases were discarded. In total, 470 cases (279 allocated to group MED and the remaining 191 cases to group XYL) were included in the final analysis.

### 3.1. Population Characteristics

Distribution of weight, age, breed, sex, ASA score, type of procedure, emergencies, and surgical positioning between both groups are shown in Table 2. Whilst both groups were similar in weight (*p* = 0.07), there was a significant difference in age (*p* = 0.002). In both groups, a comparable proportion of horses underwent repeated anaesthesia (XYL 8.9%; MED 8.6%). Procedures of multiple types during the same general anaesthesia were performed only on one horse of group XYL, which was castrated with primary intention healing and treated for crib biting by modified Forssell’s surgery (i.e., my- and neurectomy).

### 3.2. Anaesthesia

A greater percentage of horses from group XYL received acepromazine compared to MED (XYL 83.8; MED 74.9), but no significant difference in the administered doses of acepromazine could be detected (XYL 0.03 mg·kg^−1^ (0.015–0.06); MED 0.03 mg·kg^−1^ (0.004–0.06); *p* = 0.15). Acepromazine administration was repeated during anaesthesia in nine horses in group XYL and in eight horses in group MED. For sufficient sedation prior to induction, 22 horses needed less xylazine than the standard dose (0.35–1 mg·kg^−1^) and 37 horses needed more (1.15–2 mg·kg^−1^). In group MED, 15 horses needed less than the standard dose (3–6 µg·kg^−1^), and one colic horse was already profoundly sedated with xylazine and detomidine (0.28 mg·kg^−1^ and 9 µg·kg^−1^, respectively) from preceding clinical examination and did not require any sedation with medetomidine prior to anaesthesia induction. Additional top-up of medetomidine was needed in 64 horses (total dose administered: 7.5–12 µg·kg^−1^) in the MED group.

During maintenance of anaesthesia, most horses were breathing spontaneously (XYL 67.5%; MED 65.2%), and a similar proportion in each group had to be mechanically ventilated (XYL 26.2%; MED 29%). A small group of horses first showed spontaneous respiration before being switched to mechanical ventilation in the course of anaesthesia (XYL 6.3%; MED 5.7%).

In Table 3, intraoperative measures and medical treatments for both groups are presented. There was no difference in doses of administered ketamine (*p* = 0.10), thiopental (*p* = 0.22), lidocaine (*p* = 0.28), or tetrastarch (*p* = 0.61) between groups XYL and MED. Likewise, duration of tourniquet was similar (*p* = 0.51). For one horse, the duration of tourniquet was missing. Four horses of group MED received an additional CRI of ketamine (0.4–0.6 mg·kg^−1^·h^−1^). The CRIs lasted 30 to 110 min and were stopped 50 to 140 min before the end of isoflurane. Both scorers agreed on the recovery scores for these horses: 4 (twice), 3, and 2. Lidocaine CRI was administered to three horses (XYL 1; MED 2). All horses received a loading dose of 2 mg·kg^−1^ lidocaine followed by an infusion rate of 1.5–3 mg·kg^−1^·h^−1^. Administration lasted 25–75 min and was stopped 25 to 50 min before the end of anaesthesia. All three horses were given a score of 2 for recovery by both scorers. Overall, 77 horses received intraoperative local anaesthesia or analgesia. Drugs used included lidocaine, mepivacaine, ropivacaine, dexmedetomidine (combined with local anaesthetic), and morphine or a combination thereof. Routes of administration were topical via splash, intratesticular, intra-articular, perineural, or, in one instance, intravenous regional anaesthesia. In both groups, horses received additional drugs during anaesthesia (XYL 25; MED 42), namely, hydrocortisone (XYL 3; MED 13), additional morphine (XYL 4; MED 10), additional xylazine (XYL 3), atropine (XYL 1; MED 1), butylscopolamine (MED 3), flunixin (XYL 1; MED 6), full blood transfusion (XYL 1), furosemide (XYL 1), glucose (MED 1), hypertonic saline solution (MED 2), metamizole (XYL 6; MED 10), phenylbutazone (MED 1), plasma (XYL 1; MED 2), sodium bicarbonate (XYL 2), or tranexamic acid (XYL 4; MED 4).

**Figure 1:**
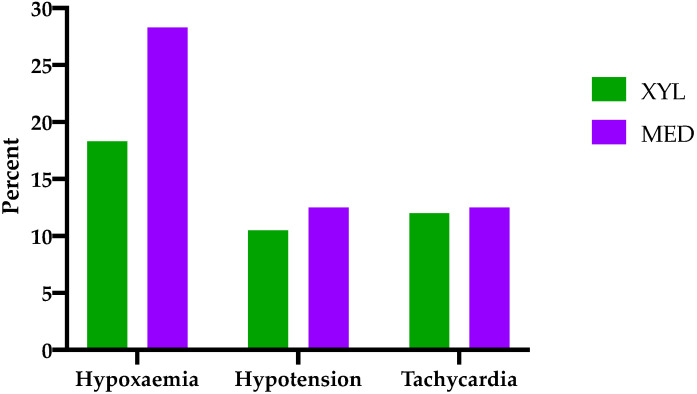
Comparison of the occurrence of intraoperative hypoxaemia, hypotension, and tachycardia between horses treated with either isoflurane–xylazine balanced anaesthesia (XYL, *n* = 191) or isoflurane–medetomidine balanced anaesthesia (MED, *n* = 279).

The occurrence of hypoxaemia, hypotension, and tachycardia is shown in Figure 1. In 18 horses, no arterial blood gas samples were available; hence, judgment of oxygenation was made on the basis of pulse oximetry. In three instances of group MED, neither arterial blood gas samples nor pulse oximetry values were available. In group MED, 13.3% of the cases were classified as hypoxaemic, and 14.7% were classified as mildly hypoxaemic. In group XYL, 6.8% were hypoxaemic, and 11.5% were mildly hypoxaemic. In six horses (XYL 3; MED 3), blood pressure was measured non-invasively, and, in five horses (XYL 2; MED 3), no blood pressure values were available. Hypotension was observed in 10.5% of the horses receiving xylazine and in 12.5% of the horses receiving medetomidine. Duration of hypotension did not significantly deviate between both groups (XYL 20 (15–100); MED 30 (15–190) min; *p* = 0.29). With regard to tachycardia, both groups showed similar occurrence (XYL 12%; MED 12.5%) and duration (XYL 30 (5–150); MED 35 (5–215) min; *p* = 0.73). Duration of anaesthesia was significantly longer in group MED compared to group XYL (155 (34–300) and 140 (45–375) min, respectively; *p* = 0.008).

### 3.3. Recovery

For recovery, two horses of the MED group received lower doses for sedation after anaesthesia (0.5 and 1.5 µg·kg^−1^), and 64 horses were administered higher doses than the standard (2.1–10 µg·kg^−1^). In the group treated with XYL, only one horse received less than the standard dose (0.26 mg·kg^−1^), and 72 horses were administered a higher dose (0.36–0.9 mg·kg^−1^). Additional medical treatment during recovery was required in 16 horses (XYL 6; MED 10) and included vitamin B supplementation (MED 1), chlorphenamine (XYL 1), dexamethasone (XYL 1), flunixin (XYL 1; MED 2), furosemide (MED 2), glucose (XYL 3; MED 6), hydrocortisone (XYL 1; MED 2), hypertonic saline solution (MED 3), lactated Ringer’s solution (XYL 1), metamizole (XYL 2; MED 1), and tetrastarch (XYL 1).

Even though, as per clinic standard, horses mostly recovered freely, in both groups, a small fraction of them were assisted during recovery (XYL 9.4%; MED 16.1%). Additionally, eight horses (XYL 2; MED 6) were too weak to get up on their own and eventually required assistance (with ropes or manually) for successful recovery. In both groups, some horses recovered with an ET tube in place (XYL 4.7%; MED 3.9%).

Table 4 summarises the recovery characteristics of both groups. Recoveries following xylazine PIVA were significantly shorter with regard to time spent in lateral recumbency (*p* < 0.001) and time to standing (*p* < 0.001), as well as attempts to reach sternal position (*p* < 0.001), but not time spent in sternal recumbency (*p* = 0.17). For two horses, attempts to reach sternal position could not be noted, since the recording started with the horse already in sternal recumbency and this information could not be retrieved from the anaesthetic protocol. In both groups, attempts to stand did not deviate significantly (*p* = 0.96).

**Figure 2:**
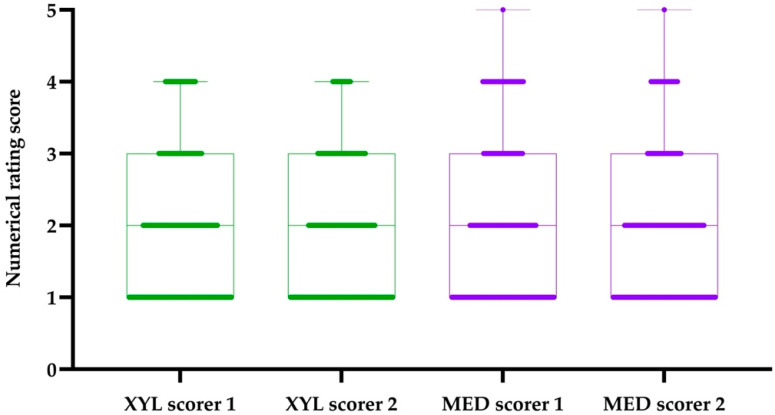
Distribution of recovery scores attributed by two independent and blinded observers (scorer 1 and scorer 2) on the basis of video recordings using a numerical rating score (1–5; 1 = smooth recovery in one attempt, 5 = recovery resulting in injury) for recoveries following xylazine partial intravenous anaesthesia (PVIA) (XYL, green) or medetomidine PIVA (MED, purple) with isoflurane. The bold horizontal lines represent the frequency of scores for each scorer and each group; the boxes imply the median, 5th–95th percentiles, and range of scores.

Using the numerical scores, the kappa test showed substantial agreement between both primary scorers (*κ* = 0.69) [19]. However, mixed-effects ordinal regression revealed that, overall, scorer 1 gave significantly higher scores (i.e., poorer recovery quality) compared to scorer 2 (*p* < 0.001), which is further visualised in Figure 2. The same analysis showed poorer recovery scores for horses and ponies needing higher doses of xylazine prior to induction (*p* = 0.01), for intraoperative administration of tetrastarch (*p* = 0.008), and for number of attempts to stand (*p* < 0.001). Overall, horses undergoing repeated anaesthesia within 6 months had significantly better recovery scores (*p* = 0.001) compared to horses undergoing general anaesthesia for the first time. Using the dichotomous recovery scores, a significant association was shown between number of attempts to stand and “bad” recovery (*p* < 0.001), confirming the results above. Furthermore, the use of salbutamol was significantly associated with poorer recovery scores (*p* < 0.001), i.e., the odds of a score of 2 decreased if salbutamol was administered. No other investigated factor (as listed in Section 2.5), including the use of MED or XYL, had any significant association with the recovery score.

Overall, both PIVA protocols produced a similar recovery quality when scores were dichotomised (XYL good: 88%, bad 12%; MED good: 83%, bad: 17%), with both groups having a median score of 2 when using the numerical rating score. One recovery in group MED resulted in a fatal cannon bone fracture following partial resection of a fractured splint bone on the same leg. The affected horse was a 5 year old warmblood mare, weighing 540 kg. Anaesthesia lasted 3 h and was uneventful (e.g., no cardiovascular or respiratory deterioration; no thiopental or ketamine boli needed). For additional analgesia, 10 mL of ropivacaine 0.75% was topically splashed on the surgical site. During recovery, the mare spent 51 min in lateral recumbency followed by 18 min in a sternal position. She took five attempts to stand and stood 69 min after the end of anaesthesia, despite the fractured leg. In the investigated population, no fatalities occurred in group XYL.

## 4. Discussion

The present retrospective study compared medetomidine vs. xylazine balanced isoflurane anaesthesia with regard to recovery quality. Overall, both treatments resulted in similarly good recoveries in the investigated population (12% “bad” recoveries (e.g., score 4 or 5) in group XYL and 17% in group MED), although times to stand took on average 14 min longer following medetomidine PIVA. Furthermore, a significant correlation between poorer recovery quality and use of higher pre-induction doses of xylazine, as well as intraoperative use of salbutamol or tetrastarch, was found. Repeated anaesthesia produced recoveries of better quality.

Recovery of horses is a multifactorial event, and, as such, it is very difficult to determine whether one PIVA regime results in better recoveries in comparison to the other. Recovery quality might be influenced by perioperatively administered drugs, duration of anaesthesia, type of surgery, intraoperative hypotension or hypoxaemia, character of the horse, presence of pain during recovery, and many other factors [8,9,20,21,22,23,24,25,26]. In the present study, factors such as age, weight, breed, sex, use of tourniquet and local anaesthesia, and position of the horse were equally distributed between the groups (the significant difference of 1.5 years was not considered to be clinically relevant).

As previously reported, horses of our study undergoing repeated general anaesthesia scored better recoveries with repetitive anaesthesia irrespective of PIVA regime used [26,27,28]. Distribution of horses with several bouts of anaesthesia was equal between both groups; therefore, it is unlikely that it influenced overall recovery quality.

Another important factor influencing recovery quality is the type of procedure. Ocular surgeries are reported to result in poorer recoveries compared to splint bone surgeries [29], as well as show longer duration recoveries [30]. The present report investigated a similar proportion of ocular surgeries in both groups without any influence on recovery scores. Unfortunately, group MED reflected a higher percentage of emergency laparotomy surgeries with sicker horses (i.e., more ASA IV horses in group MED) and longer anaesthesia times, all facts known to usually impair recovery quality [31], particularly in comparison to elective surgeries in dorsal recumbency [32]. Despite this, only the few factors listed at the beginning of the discussion were shown to have a significant effect on recovery, and it is interesting that horses in the MED group did not get up worse than those in the XYL group.

There is evidence that intraoperative hypoxaemia might prolong recovery [31,33] and have a negative effect on recovery quality [25], particularly in horses undergoing emergency laparotomy due to colic where intraoperative hypoxaemia increased fatality rate [31]. The present study did not find that intraoperative hypoxaemia affected recovery score, but the use of intraoperative salbutamol did. It is possible that, in these horses, hypoxaemia during recovery reoccurred. Together with the great increase in oxygen demand during recovery [34], oxygenation might have become insufficient during a period where the horse was regaining consciousness, thus impacting the quality of recovery.

Despite the fact that horses in group MED spent on average 10 min longer in lateral recumbency, this did not result in a difference in recovery quality when using the in-house RQSS. The present study cannot determine whether this difference in duration of lateral recumbency and time to stand is solely attributed to a drug effect, as the MED group represented a higher percentage of emergency laparotomies with prolonged anaesthesia times, which is known to be associated with poorer recovery quality [31,32]. However, in the present study, ASA score did not affect the overall quality of recovery, whereas the use of tetrastarch did. Tetrastarch was used in cases where hypovolaemia was suspected and the use of a crystalloid bolus was not successful in establishing normal circulation within predetermined clinically acceptable ranges. It is likely that those horses suffered from suboptimal tissue perfusion and oxygenation during anaesthesia, resulting in weakness and, therefore, worse recovery quality. In a clinical setting, the exact assessment of tissue perfusion and oxygenation is unfortunately still impossible. A recent clinical and well-standardised study comparing healthy horses undergoing elective surgery with medetomidine or xylazine PIVA did not find a difference in oxygenation between both groups, but minimally higher mean arterial blood pressures with xylazine, despite a lower mean dose of dobutamine (medetomidine 36 µg·kg^−1^·h^−1^, xylazine 24 µg·kg^−1^·h^−1^) [18]. In the present study, incidence of hypotension was similar between groups, despite the fact that, in the MED group, fewer horses received acepromazine, probably as a consequence of more laparotomy surgeries in that group. It is difficult to judge the significance of those findings, as perfusion and tissue oxygenation are rather related to cardiac output than simply to mean arterial blood pressure [35]. Further experimental randomised crossover studies with extensive cardiopulmonary monitoring are warranted to elucidate this topic.

The mean duration of anaesthesia in group MED was 15 min longer compared to XYL. It is known that longer anaesthetic times result in poorer recovery scores [21,30] and a higher incidence of fatal outcome [36]. Interestingly, studies including alpha-2 adrenergic agonist PIVA did not show this effect [14,25,37]. In this light, it seems unlikely that the mean difference of only 15 min anaesthesia time affected the recoveries in this study, particularly as mean anaesthesia time in both groups was considerable (XYL 140 and MED 155 min). Experimental studies have shown that a longer duration of recovery generally results in better recovery scores [37]. In the current study, xylazine recoveries were shorter without a difference in quality which was also shown by other authors more recently [25,38]. As prolonged lateral recumbency may have detrimental impacts on ventilation, muscle and nerve perfusion, and their concurrent function [39], xylazine may be more suitable for PIVA in horses because the recovery duration is shorter but still of good quality.

The temperament of horses is a known factor to influence recovery quality [37]. In the present report, a higher dose of xylazine necessary for preoperative sedation was associated with poorer recovery quality. This should not imply that lower doses of xylazine should be used preoperatively to have better recoveries, but more that probably worse recoveries should be anticipated in fractious horses that need high doses of sedatives prior to anaesthesia induction.

To allow retrospective evaluation of recoveries, DVD recordings of the recoveries were used. This allowed for the recovery to be scored by several independent observers unaware of group allocation, duration of anaesthesia, or other possible relevant factors, and the videos could be edited to exclude prolonged phases of inactivity and, therefore, prevent scorer fatigue. On the downside, audio was not recorded for protection of privacy, potentially confounding the rating of recovery by making it difficult for the observers to appraise the severity of an impact when horses crashed into the walls or on the ground.

A multitude of RQSSs have been developed and are routinely used for assessment of recovery quality, including visual analogue scales (VAS), numerical scores, and composite scores. Lacking a gold-standard RQSS, several studies have previously evaluated the reliability of different scores [40,41,42]. Composite scoring systems, in which multiple aspects of the recovery are ranked and their numbers added or even used in a formula to calculate a score, are said to be the more accurate, but also more time-consuming in their application [42,43]. Suthers et al., who evaluated three different scoring systems, found that practicality of use seemed to be inextricably tied to imprecision [42]. Vettorato et al. found similar agreement among the four RQSSs tested and suggested that the choice of recovery score should be rather based on the applicability [41]. The clinic’s in-house numerical scoring system was used for the present study, because of its simple application, the familiarity of all observers with this particular score, and the fact that the power analysis of the present study was based on a study using the same scoring system [18]. Even though this scoring system has not yet been validated, it has previously been applied in many other studies [18,25,37,44]. Menzies et al. used this same score alongside a VAS and found excellent agreement between both scores [44]. VAS on the other hand has been shown to have similar reliability compared to numerical and composite RQSSs [41].

To limit individual scoring inaccuracies, two independent scorers assessed recoveries and, where agreement between both scorers was poor, two senior clinicians re-evaluated the recordings. Despite some horses with poor scorer agreement, the data used for analysis can be considered reliable, since recoveries with very poor agreement were eliminated. Furthermore, a great number of horses were included in this study, which mitigated effects of outliers. It is noteworthy that some intermediate quality recoveries were given a different score by each observer, which suggests that objective scoring of certain recoveries is difficult and may depend on personal expectations and experience. This also stands in agreement with the findings of Vettorato et al., who showed a greater variability of the intermediate quality recoveries when investigating the reliability of four different RQSSs [41]. However, the influence of observer experience on the grading of recoveries was previously investigated by Farmer et al. using a VAS, and no effect of observer experience on the scoring was found [45].

The fatal outcome of one horse treated with MED is unlikely to be related to the alpha-2 adrenergic agonist used. The horse had suffered from a chronically infected splint bone fracture for 3 weeks prior to surgery. Even though X-rays of the affected leg prior to surgery, as well as intraoperative X-rays after fragment removal, were unremarkable, it is possible that a pre-existing fissure of the cannon bone remained unnoticed and fractured under the impacts of recovery. It was also discussed if the ropivacaine splashed on the surgical site could have diffused to adjacent nerves, leading to deficits in proprioception and, consequently, inappropriate limb placement and fracture of the malpositioned leg.

The power calculation performed prior to this study was based on numbers of a clinical study performed in elective cases. The number calculated was valid for an even distribution of comparable cases in each group. The herewith investigated population is very heterogeneous and may not be comparable. More horses would be necessary for a clearer result and to even out potential influence of other factors affecting recovery. The results of the current study are only applicable to the specific clinic, population, and peri-anaesthetic management practised at the institution where the investigation took place.

Repeating the power calculation with the incidence of bad recoveries from this study, 1554 recoveries (777 per group) would be necessary to conclusively investigate whether the found incidences are true and that there really are no differences in recovery quality between isoflurane PIVA with medetomidine vs. xylazine.

This study faced another three limitations. First, the retrospective nature inevitably made it impossible to have a standardised population with regard to demographics, procedure, and anaesthetic protocol. Second, whether the horses received MED or XYL was decided on the basis of their registration status as either a food-producing animal (XYL) or a non-food-producing animal (MED), rather than on a randomised allocation. This led to a ratio of 60:40% (MED:XYL), instead of an even distribution between both protocols. However, the anaesthetic management at this clinic is well standardised (use of perioperative drugs, management of cardiopulmonary function, and management of recovery). To warrant an equal ratio, a prospective, randomised study design would have been favourable; however, due to the amount of recovery required, this is not practical to obtain timely results. Last but not least, no preoperative pain scoring was performed, and the influence of pre-existing pain on the results could not be tested.

## 5. Conclusions

According to the results of this study, there is no increased risk in the occurrence of a potentially dangerous recovery when using medetomidine balanced isoflurane anaesthesia compared to xylazine. However, more prospective trials including large numbers of recoveries are warranted.

## Figures and Tables

**Table 1:** Numerical recovery quality scoring system used for grading the recoveries following general anaesthesia under partial intravenous anaesthesia with either xylazine or medetomidine and isoflurane.

Score	Definition
1	Standing successfully at the first attempt.
2	Standing successfully at the second attempt.
3	More than two attempts to stand, but horse remains calm.
4	Several attempts to stand; apparent risk of injury.
5	Horse injured during recovery.

**Table 2:** Distribution of body weight (median (range)), age (median (range)), breed, sex, American Society of Anaesthesiologists (ASA) score, procedure, emergencies, and surgical positioning between two groups of horses undergoing partial intravenous anaesthesia using isoflurane with either xylazine (XYL, *n* = 191) or medetomidine (MED, *n* = 279). AH = American horse, BH = Baroque horse, DH = draught horse, IH = Icelandic horse, SDH = small draught horse, TB = Thoroughbred, WB = warmblood. LAP = laparotomy, ORT = orthopaedics, OFX = fractures, OPHT = ophthalmologic procedures, VISC = visceral surgeries, such as castrations and skin tumour excisions, ENT = surgeries on the head and throat, excluding ophthalmologic procedures, MULT = multiple procedures. RL = right-lateral recumbency, LL = left-lateral recumbency, DR = dorsal recumbency, CH = changing positioning during procedure.

	XYL	MED
**Body weight (kg)**	510 (200–740)	520 (200–740)
**Age (years)**	10.4 (0.3–29.8)	11.9 (0.3–26.3)
**Breed %**		
AH	5.8	6.8
Arabian	3.1	5.4
BH	9.4	9.7
DH	0.5	0.7
IH	10.5	11.1
Pony	5.8	5
SDH	13.6	7.2
TB	1	2.9
Unknown	1	1.1
WB	49.2	50.2
**Sex %**		
Female	34.6	39.1
Gelding	36.6	48.4
Male	28.8	12.5
**ASA score %**		
ASA I	17.8	8.2
ASA II	63.4	59.9
ASA III	9.9	11.5
ASA IV	7.9	18.6
ASA V	1	1.8
**Procedure %**		
LAP	10.5	25.1
ORT	35.1	35.5
OFX	6.3	7.2
OPHT	9.4	12.2
VISC	30.4	13.6
ENT	7.9	6.5
MULT	0.5	0
**Emergency %**	18.3	39.4
**Positioning %**		
RL	19.9	19.7
LL	18.8	21.5
DR	58.1	57.3
CH	3.1	1.4

**Table 3:** Distribution of intraoperative measures and medical treatment for horses undergoing general anaesthesia with either isoflurane–xylazine partial intravenous anaesthesia (PIVA) (XYL, *n* = 191) or isoflurane–medetomidine PIVA (MED, *n* = 271). Data are presented as median (range). Local Ax = local anaesthesia and/or analgesia; NMBA = neuromuscular blocking agent.

	XYL	MED
**Ketamine**		
Percent %	61.8	56.6
Dose (mg·kg^−1^·h^−1^)	0.09 (0.01–0.96)	0.08 (0.02–0.65)
**Thiopental**		
Percent %	35.1	26.2
Dose (mg·kg^−1^·h^−1^)	0.3 (0.07–5.56)	0.2 (0.07–1.11)
**Lidocaine Bolus**		
Percent %	6.3	6.8
Dose (mg·kg^−1^)	2 (1–5.6)	2 (1–4)
**Tetrastarch**		
Percent %	8.9	12.5
Dose (mL·kg^−1^)	3.5 (0.8–7.7)	3.5 (1–9.6)
**Salbutamol %**	7.3	18.3
**Polymyxin B %**	6.8	13.6
**Local Ax %**	29.3	17.9
**NMBA %**	1	3.6
**Tourniquet**		
Percent %	14.1	8.6
Duration (min)	105 (45–190)	97.5 (5–150)

**Table 4:** Time spent in lateral and sternal recumbency, time to stand, number of attempts to sternal position, and number of attempts to stand following general anaesthesia with isoflurane partial intravenous anaesthesia using either xylazine (XYL, *n* = 191) or medetomidine (MED, *n* = 279). Results are presented as median (range).

Recovery Characteristic	XYL	MED	*p*
Lateral recumbency (min)	35 (3–138)	45 (9–124)	<0.001
Sternal recumbency (min)	7 (0–66)	10 (0–70)	0.17
Time to stand (min)	43 (13–175)	57 (13–155)	<0.001
Attempts to sternal	1 (1–14)	2 (1–14)	<0.001
Attempts to stand	2 (1–12)	2 (1–14)	0.96

## Data Availability

Publicly available datasets were analysed in this study. These data can be found at https://bit.ly/2RpElWP, accessed on 16 May 2021.
